# Effect of different levels of bioplex manganese along with probiotics and multi‐enzymes on performance and immune system indices of broilers

**DOI:** 10.1002/vms3.479

**Published:** 2021-05-01

**Authors:** Mohammad Javad Babaei, Jafar Fakhraei, Hossein Mansoori Yarahmadi, Masoud Gomarian

**Affiliations:** ^1^ Department of Animal Science Islamic Azad University Arak Iran

**Keywords:** bioplex manganese, broiler, immune system, performance, probiotics

## Abstract

To investigate the effect of different levels of bioplex manganese along with probiotics and multi‐enzymes on the performance and immune system of broilers, 640 one‐day‐old male chicks of the Ross 308 strain were reared and the data analysed in a 4 × 2 × 2 factorial experiment with four levels of bioplex manganese (0, 60, 72 and 84 mg per kg of diet), two levels of Parsilact probiotic (0 and 200 mg per kg of diet) and two levels of Combo multi‐enzyme (0 and 1,000 mg per kg of diet) in a completely randomized design with 16 experimental treatments, 4 replicates and 10 chickens per replicate during a period of 42 days. The results showed that the performance of the broiler chickens in the diets containing 72 and 84 mg bioplex manganese along with probiotics and multi‐enzymes had the greatest difference compared to the control (*p* < .05). Compared to the control with 0 mg/kg manganese; the bursa of Fabricius weight was greater in chickens fed diets containing additional manganese (*p* < .05). The concentration of antibodies produced against Newcastle disease virus, as well as the concentrations of IgG, IgM and total immunoglobulins produced against SRBC, were highest in the group fed a diet containing 84 mg manganese along with probiotics and multi‐enzymes (*p* < .05). The results show combining additional manganese with probiotics and multi‐enzymes in chicken diets leads to better performance as well as a stronger immune system of chickens.

## Abstract 2

The results show combining additional manganese with probiotics and multi‐enzymes in chicken diets leads to better performance as well as a stronger immune system of chickens.

## 1 INTRODUCTION

As the highest cost of poultry production is the cost of feed, optimizing feed consumption and reducing feed conversion ratio are very important in the poultry industry (Ahmadi et al., 2018). The efficiency of the digestion and absorption process in poultry is directly related to dietary compounds and microorganisms located in their digestive system. Therefore, several food additives have been developed to improve the diets and gastrointestinal conditions of poultry (Poorghasemi et al., 2013). Such additives often include trace elements (Ahmadi et al., 2019).

Manganese is a key trace element in poultry which plays a very important role in the body's reactions, especially those related to bone and cartilage formation. It is also essential for the prevention of oxidative stress, enzyme activities and metabolism of amino acids, carbohydrates and cholesterol. Suppn has been shown to have a positive effect on the growth performance and immune system of broilers (Ji et al., 2006). Manganese is usually provided in the diet of broilers in the form of inorganic minerals (mostly oxides and sulphates), but organic chelated (biolplex) forms of manganese are also available (Fly et al., 1989). Bioplexes are organic complexes (principally protein‐based) of metallic elements. They have a higher rate of absorption in the intestine than inorganic forms (Berta et al., 2004) and their use thus can reduce the required dietary concentration of some minerals, thereby reducing without affecting productivity or product safety (Virden et al., 2003).

Other potential feed additives include probiotics and enzymes. Probiotics are used to increase the growth of beneficial microbes in the digestive system and to eliminate pathogens in poultry feed. Probiotics can also have a positive effect on the immune system and production efficiency (Thorat et al., 2015), thereby reducing disease and improving feed conversion ratio without resulting in tissue residues or, unlike antibiotics, causing microbial resistance (Poorghasemi et al., 2017).

Enzymes reduce the quality and quantity of available substrate for microbes, decrease the viscosity of intestinal contents and increase the rate of substance transmission by increasing the rate of digestion and production of simple sugars from diet fibre, thus reducing the harmful microbial population of the intestine. Enzymes also reduce fermentation in the intestine and increase fermentation in the cecum by increasing the digestibility of dietary nutrients (Ghomi et al., 2012; Sun & Kim, 2019). Some substances digested by gastrointestinal enzymes may not be absorbed in the small intestine and be transferred to the cecum, thus increasing bacterial fermentation in the cecum (Ghomi et al., 2012). It has been reported that enzyme supplements can increase the growth performance of poultry by up to 10% (Daghigh Kia et al., 2017). Like probiotics, some of this may be mediated by improvements in immune function (Thorat et al., 2015). Combining enzymes and probiotics may be beneficial; Midilli (2001) stated that enzymes can indirectly prevent the proliferation of harmful bacteria by providing a suitable environment for digestion, and in such conditions, probiotics can lead to more production of useful microbes.

Numerous results from past research have shown that manganese, probiotics and enzymes in broiler diets have positive effects on the performance and immune system of broilers. As these compounds are rarely used in combination in the diets of broilers and on the other hand, the double beneficial effects of the simultaneous use of manganese, probiotics and enzymes can be important in terms of nutritional innovation in broiler breeding, the present experiment was performed to investigate the main effects of manganese, probiotics and multi‐enzymes and their interaction on the performance and immune system of broilers.

## 2 MATERIALS AND METHODS

This research was conducted under the principles of animal research ethics in the city of Somehsara, Iran (37.2071°N, 50.0034°E).

In this study, 640 one‐day‐old male chickens of Ross 308 strain were used in a 4 × 2 × 2 factorial experiment with four levels of bioplex manganese (0, 60, 72 and 84 mg per kg of diet), two levels of Parsilact probiotic (0 and 200 mg per kg of diet) and two levels of Combo multi‐enzymes (0 and 1,000 mg per kg of diet) in a completely randomized design with 16 experimental treatments, 4 replicates and 10 chickens per replicate during a period of 42 days.

The experimental diets included the starter (1–21 days of age) and finisher (22–42 days of age) diets, which were adjusted, based on the handbook of Ross 308 strain requirements, using UFFDA software (Table 1). During the breeding period, all the groups received the same diets based on maize‐soybean. The experimental treatments include: 1‐ control (without additives), 2‐ control + probiotics (200 mg per kg of diet), 3‐ control + multi‐enzyme (1,000 mg per kg of diet), 4‐ control + probiotics (200 mg per kg of diet) + multi‐enzyme (1,000 mg per kg of diet), 5‐ control + manganese (60 mg per kg of diet), 6‐ control + manganese (60 mg per kg of diet) + probiotics (200 mg per kg of diet), 7‐ control + manganese (60 mg per kg of diet) + multi‐enzyme (1,000 mg per kg of diet), 8‐ control + manganese (60 mg per kg of diet) + probiotics (200 mg per kg of diet) + multi‐enzyme (1,000 mg per kg of diet), 9‐ control + manganese (72 mg per kg of diet), 10‐ control + manganese (72 mg per kg of diet) + probiotics (200 mg per kg of diet), 11‐ control + manganese (72 mg per kg of diet) + multi‐enzyme (1,000 mg per kg of diet), 12‐ control + manganese (72 mg per kg of diet) + probiotics (200 mg per kg of diet) + multi‐enzyme (1,000 mg per kg of diet), 13‐ control + manganese (84 mg per kg of diet), 14‐ control + manganese (84 mg per kg of diet) + probiotics (200 mg per kg of diet), 15‐ control + manganese (84 mg per kg of diet) + multi‐enzyme (1,000 mg per kg of diet), 16‐ control + manganese (84 mg per kg of diet) + probiotics (200 mg per kg of diet) + multi‐enzyme (1,000 mg per kg of diet).

**TABLE 1:** The ingredients of the experimental diets

Ingredients (%)	Starter (1–21 days old)	Grower (22–42 days old)
Corn	49.94	58.59
Soybean meal	40.00	33.00
Soybean oil	6.10	5.00
Calcium bicarbonate	1.25	1.37
Dicalcium phosphate	1.92	1.39
Salt	0.30	0.25
Mineral premix^a^	0.25	0.25
Vitamin premix^b^	0.25	0.25
DL‐methionine	0.14	0.05
Chemical analyses		
Metabolizable energy (kcal/kg)	3,196	3,184
Crud protein (DM)	23.00	20.00
Lysine (%)	1.10	1.00
Methionine + Cysteine (%)	0.90	0.72
Methionine (%)	0.50	0.38
Threonine (%)	0.80	0.74
Ca (%)	1.20	1.00
Available Phosphorus (%)	0.45	0.35
Cl (%)	0.18	0.17

^a^ Mineral mixture per ton of diet: Mn: 100 mg; Zn: 100 mg; Fe: 50 mg; Cu: 10 mg; Se: 0.2 mg and I: 1 mg.

^b^ Vitamin premix per ton of diet: vitamin A: 900 IU; vitamin D_3_: 2000 IU; vitamin E: 18 IU; vitamin K_3_: 2 mg; vitamin B_6_: 3 mg; vitamin B_12_: 0.015 mg; Thiamine: 1.8 mg; Riboflavin: 6.6 mg; Pantothenate: 10 mg; Nicotinic acid: 30 mg; Folic acid: 1 mg; vitamin H2: 0.1 mg and Choline chloride: 500 mg.

The bioplex manganese used in the present experiment was prepared from Alltech lnc Nicholasville, USA, and also the probiotic used under the brand Parsilact was prepared from the biological products company of Pardis Roshd Mehregan Iran, which contained 2.3 × 10^11^ CFU/g of bacteria. The multi‐enzyme under the brand Combo was a product of the American Bio‐system company, one gram of which contained 1,000 units of phytase, 75 units of cellulase, 55 units of xylanase and 50 units of beta‐glucanase.

## 3 PERFORMANCE

During the study period, breeding conditions were the same for all chickens and feed was available ad‐lib. The feeds in each replicate were weighed before consumption and by specifying the weight of the remaining feed at the end of each week, the amount of feed consumed for each treatment for the entire period was calculated. Chickens were weighed weekly and the weight gain and conversion ratio of the entire period was determined (Poorghasemi et al., 2017).

## 4 HUMORAL IMMUNITY

### 4.1 Determination of antibody titre produced against Newcastle disease virus

To determine antibody response to Newcastle disease virus vaccination, Newcastle disease vaccine was injected at 22 days of age, and birds blood sampled was pe10 and 20 days later. For blood sampling, two birds were randomly selected from each replicate and 2 ml of blood was taken from the wing vein. Blood samples were transferred to the laboratory and centrifuged for 15 min at 3,000 rpm to extract serum. Antibody titre was determined by agglutination inhibition (HI) assay (Poorghasemi et al., 2015).

### 4.2 Determination of the titres of the immunoglobulins produced against SRBC

To measure the titre of immunoglobulins produced against sheep red blood cells (SRBC) on 28 and 35 days of age, 4 chickens per experimental unit were injected with 0.2 ml of SRBC suspension (5%) washed in sterile phosphate buffer, in the chest muscle. Seven days after each injection, on days 35 and 42 of age, about 2 ml of blood was drawn from the same birds (marked with colour on the feathers) from the wing vein. Blood samples were kept at laboratory temperature for 6 hr to separate the serum from the blood clot. The obtained serum was then centrifuged at 4,000 rpm for 15 min. The microtitre haemagglutination method was then used to determine IgM and IgG titres as well as the total titres of immunoglobulins produced against SRBC (Poorghasemi et al., 2015).

### 4.3 Statistical analysis

Data were analysed using SAS statistical software Release 9.4. (SAS Institute, 2004). A general linear model was used: Y_ijkl_ = μ + A_i_ + B_j_ + C_k_ + (AB)_ij_ + (AC)_ik_ + (BC)_jk_ + (ABC)_ijk_ + e_ijkl_. In this model, Y_ijkl_ was the value of each observation for the studied trait, µ was the average of observations, A_i_ was the manganese effect, B_j_ was the probiotic effect, C_k_ was the multi‐enzyme effect, (AB)_ij_ was the effect of the combined use of manganese and probiotic, (AC)_ik_ was the effect of the combined use of manganese and multi‐enzymes, (BC)_jk_ was the effect of the combined use of probiotics and multi‐enzymes, (ABC)_ijk_ was the effect of the combined use of manganese, probiotics and multi‐enzymes and e_ijkl_ was the effect of experimental error. Where the model was found to have a *p*‐value < .05, means of treatments were compared using Duncan's test to account for the multiple comparisons.

## 5 RESULTS

The results on the effect of experimental treatments on the performance of broilers are presented in Table 2. Weight gain during the entire period showed a significant difference between different levels of manganese (*p* < .05) such that the highest rate of weight gain was related to 84 mg of manganese and the lowest rate was related to the control. With diets containing probiotics and multi‐enzymes, the weight gain was significantly higher as compared to the control (*p* < .05). Weight gain with the combined use of manganese (72 and 84 mg), probiotics and multi‐enzymes had the highest significant difference compared to the control (*p* < .05). Feed intake for the entire period did not show a significant difference between different levels of manganese, probiotics, multi‐enzymes and their combined uses (*p* > .05). The feed conversion ratio was significantly lower than the control when using different levels of manganese as well as probiotics and multi‐enzymes (*p* < .05). In the interacting effects of the combined uses, the conversion ratio of the diets containing 72 and 84 mg manganese with probiotics and multi‐enzymes had the lowest significant difference compared to the control (*p* < .05).

**TABLE 2:** The effects of experimental diets on body weight gain (BWG), feed intake (FI) and feed conversion ratio (FCR)

Treatment							BWG (g/hen/period)	FI (g/hen/period)	FCR
Mn (mg/kg diet)					0		58.242^b^	117.375	2.018^a^
					60		61.286^a^	119.265	1.949^b^
					72		61.322^a^	118.049	1.940^b^
					84		62.338^a^	119.240	1.914^b^
*SEM*							0.493	0.699	0.017
*p*‐value							<0.0001	0.1651	0.0007
Probiotic (mg/kg diet)					0		60.035^b^	118.798	1.982^a^
					200		61.559^a^	118.166	1.928^b^
*SEM*							0.349	0.495	0.012
*p*‐value							0.0033	0.3708	0.0028
Multi‐enzyme (mg/kg diet)					0		59.978^b^	118.933	1.987^a^
					1,000		61.616^a^	118.032	1.924^b^
*SEM*							0.349	0.495	0.012
*p*‐value							0.0017	0.2039	0.0006
Mn (0 mg/kg diet) ‐ P (0 mg/kg diet) ‐ ME (0 mg/kg diet)							56.297^e^	117.983	2.096^a^
Mn (0 mg/kg diet) ‐ P (200 mg/kg diet) ‐ ME (0 mg/kg diet)							60.144 cd	118.207	1.966^bcde^
Mn (0 mg/kg diet) ‐ P (0 mg/kg diet) ‐ ME (1,000 mg/kg diet)							58.962^de^	118.889	2.019^abc^
Mn (0 mg/kg diet) ‐ P (200 mg/kg diet) ‐ ME (1,000 mg/kg diet)							62.668^bc^	119.499	1.907^cde^
Mn (60 mg/kg diet) ‐ P (0 mg/kg diet) ‐ ME (0 mg/kg diet)							56.696^e^	115.330	2.035^ab^
Mn (60 mg/kg of diet) ‐ P (200 mg/kg of diet) ‐ ME (0 mg/kg diet)							60.996 cd	119.101	1.955^bcde^
Mn (60 mg/kg diet) ‐ P (0 mg/kg diet) ‐ ME (1,000 mg/kg diet)							58.926^de^	118.863	2.018^abc^
Mn (60 mg/kg diet) ‐ P (200 mg/kg diet) ‐ ME (1,000 mg/kg diet)							63.354^bc^	120.998	1.912^cde^
Mn (72 mg/kg diet) ‐ P (0 mg/kg diet) ‐ ME (0 mg/kg diet)							56.709^e^	119.204	2.104^a^
Mn (72 mg/kg diet) ‐ P (200 mg/kg diet) ‐ ME (0 mg/kg diet)							60.895 cd	118.755	1.950^bcde^
Mn (72 mg/kg diet) ‐ P (0 mg/kg diet) ‐ ME (1,000 mg/kg diet)							59.144^de^	118.235	2.002^abcd^
Mn (72 mg/kg diet) ‐ P (200 mg/kg diet) ‐ ME (1,000 mg/kg diet)							64.326^b^	120.341	1.873^e^
Mn (84 mg/kg diet) ‐ P (0 mg/kg diet) ‐ ME (0 mg/kg diet)							60.829 cd	117.952	1.939^bcde^
Mn (84 mg/ mg/kg diet) ‐ P (200 mg/kg diet) ‐ ME (0 mg/kg diet)							61.840^bcd^	120.156	1.944^bcde^
Mn (84 mg/kg diet) ‐ P (0 mg/kg diet) ‐ ME (1,000 mg/kg diet)							61.492^bcd^	116.307	1.893^de^
Mn (84 mg/kg diet) ‐ P (200 mg/kg diet) ‐ ME (1,000 mg/kg diet)							69.474^a^	115.895	1.670^f^
*SEM*							0.987	1.399	0.034
*p*‐value							<0.0001	0.2649	<0.0001

Note

ME: Multi‐enzyme; P: probiotic; *SEM*: standard error of the means.

The means within the same column with at least one common letter, do not have significant difference *p* > .05)

^a, b, c, d, e, f^ the means within the same row with different letter, are significantly different (*p* < 0.05).

Table 3 shows the effect on the lymphoid organs of broilers at 42 days of age. The weights of thymus and spleen in different levels of manganese did not show a significant difference (*p* > .05) but bursa weight significantly increased in all levels of manganese compared to the control (*p* < .05). Although the use of probiotics and multi‐enzymes in broiler diets did not make a significant difference in the weight of lymphoid organs compared to the control, their weights were numerically higher than the control (*p* > .05). Regarding the combined uses of manganese, probiotics and multi‐enzymes on the weight of thymus, spleen and bursa, the results showed that the diets containing different levels of manganese with probiotics and multi‐enzymes had the highest difference with the control but this difference was not significant (*p* > .05).

**TABLE 3:** The effects of experimental diets on weight of lymphoid organs

Treatment		Thymus (g)	Spleen (g)	Bursa (g)
Mn (mg/kg diet)	0	5.875	4.255	2.469^b^
	60	5.885	4.816	3.175^a^
	72	6.221	5.215	3.561^a^
	84	6.641	5.254	3.599^a^
*SEM*		0.627	0.245	0.413
*p*‐value		0.8021	0.0066	0.2972
Probiotic (mg/kg diet)	0	6.13	3.142	4.664
	200	6.182	3.261	5.106
*SEM*		0.444	0.173	0.292
*p*‐value		0.9344	0.6295	0.2904
Multi‐enzyme (mg/kg diet)	0	5.933	3.165	4.707
	1,000	6.378	3.238	5.064
*SEM*		0.444	0.173	0.292
*p*‐value		0.4809	0.7682	0.3914
Mn (0 mg/kg diet) ‐ P (0 mg/kg diet) ‐ ME (0 mg/kg diet)		5.245	2.17	3.045
Mn (0 mg/kg diet) ‐ P (200 mg/kg diet) ‐ ME (0 mg/kg diet)		6.692	3.212	5.44
Mn (0 mg/kg diet) ‐ P (0 mg/kg diet) ‐ ME (1,000 mg/kg diet)		5.302	2.7	4.105
Mn (0 mg/kg diet) ‐ P (200 mg/kg diet) ‐ ME (1,000 mg/kg diet)		6.3	3.397	4.875
Mn (60 mg/kg diet) ‐ P (0 mg/kg diet) ‐ ME (0 mg/kg diet)		6.66	3.507	4.653
Mn (60 mg/kg of diet) ‐ P (200 mg/kg of diet) ‐ ME (0 mg/kg diet)		5.482	3.167	5.16
Mn (60 mg/kg diet) ‐ P (0 mg/kg diet) ‐ ME (1,000 mg/kg diet)		5.577	3.702	3.933
Mn (60 mg/kg diet) ‐ P (200 mg/kg diet) ‐ ME (1,000 mg/kg diet)		6.778	3.85	5.59
Mn (72 mg/kg diet) ‐ P (0 mg/kg diet) ‐ ME (0 mg/kg diet)		6.617	2.66	4.845
Mn (72 mg/kg diet) ‐ P (200 mg/kg diet) ‐ ME (0 mg/kg diet)		5.47	2.347	3.59
Mn (72 mg/kg diet) ‐ P (0 mg/kg diet) ‐ ME (1,000 mg/kg diet)		5.947	3.73	3.908
Mn (72 mg/kg diet) ‐ P (200 mg/kg diet) ‐ ME (1,000 mg/kg diet)		7.077	3.868	6.087
Mn (84 mg/kg diet) ‐ P (0 mg/kg diet) ‐ ME (0 mg/kg diet)		5.465	2.825	5.12
Mn (84 mg/ mg/kg diet) ‐ P (200 mg/kg diet) ‐ ME (0 mg/kg diet)		4.837	3.662	5.185
Mn (84 mg/kg diet) ‐ P (0 mg/kg diet) ‐ ME (1,000 mg/kg diet)		6.61	2.482	5.395
Mn (84 mg/kg diet) ‐ P (200 mg/kg diet) ‐ ME (1,000 mg/kg diet)		8.428	3.937	7.233
*SEM*		1.255	0.489	0.826
*p*‐value		0.9122	0.1548	0.1263

Note

ME: Multi‐enzyme; P: probiotic; *SEM*: standard error of the means.

The means within the same column with at least one common letter, do not have significant difference (*p* > .05)

^a, b, c, d, e, f^ the means within the same row with different letter, are significantly different (*p* < 0.05).

The results on the levels of antibody titres produced against Newcastle disease virus (NDV) are shown in Table 4. Various levels of manganese as well as the use of probiotics did not cause a significant difference in any of the stages on the anti‐NDV antibodies (*p* > .05). However, the antibody titre in the first stage in the diet containing multi‐enzyme had a significant increase compared to the control (*p* < .05). In the combined use, the antibody titre produced against Newcastle in the first stage was significantly increased for the groups fed with 72 and 84 mg manganese with probiotics and multi‐enzymes compared to the control (*p* < .05). The combined uses of different levels of manganese, probiotics and multi‐enzymes on the antibody titre produced against Newcastle in the second stage was not significant (*p* > .05).

**TABLE 4:** The effects of experimental diets on immune responses of broiler chickens after vaccination

Treatment		ATAN1 (log_2_)	ATAN2 (log_2_)
Mn (mg/kg diet)	0	3.313	5
	60	3.25	5.25
	72	2.938	5.813
	84	3.5	5.375
*SEM*		0.163	0.302
*p*‐value		0.1195	0.2975
Probiotic (mg/kg diet)	0	3.188	5.313
	200	3.313	5.406
*SEM*		0.116	0.214
*p*‐value		0.448	0.7579
Multi‐enzyme (mg/kg diet)	0	3.000^b^	5.375
	1,000	3.500^a^	5.344
*SEM*		0.116	0.214
*p*‐value		0.0036	0.9181
Mn (0 mg/kg diet) ‐ P (0 mg/kg diet) ‐ ME (0 mg/kg diet)		2.500^c^	4
Mn (0 mg/kg diet) ‐ P (200 mg/kg diet) ‐ ME (0 mg/kg diet)		3.500^abc^	5.75
Mn (0 mg/kg diet) ‐ P (0 mg/kg diet) ‐ ME (1,000 mg/kg diet)		3.500^abc^	4.5
Mn (0 mg/kg diet) ‐ P (200 mg/kg diet) ‐ ME (1,000 mg/kg diet)		3.250^abc^	5.75
Mn (60 mg/kg diet) ‐ P (0 mg/kg diet) ‐ ME (0 mg/kg diet)		3.500^abc^	5.25
Mn (60 mg/kg of diet) ‐ P (200 mg/kg of diet) ‐ ME (0 mg/kg diet)		2.500^c^	5.25
Mn (60 mg/kg diet) ‐ P (0 mg/kg diet) ‐ ME (1,000 mg/kg diet)		3.500^abc^	5.5
Mn (60 mg/kg diet) ‐ P (200 mg/kg diet) ‐ ME (1,000 mg/kg diet)		3.500^abc^	5
Mn (72 mg/kg diet) ‐ P (0 mg/kg diet) ‐ ME (0 mg/kg diet)		3.000^bc^	5.75
Mn (72 mg/kg diet) ‐ P (200 mg/kg diet) ‐ ME (0 mg/kg diet)		2.500^c^	5.75
Mn (72 mg/kg diet) ‐ P (0 mg/kg diet) ‐ ME (1,000 mg/kg diet)		3.000^c^	5.75
Mn (72 mg/kg diet) ‐ P (200 mg/kg diet) ‐ ME (1,000 mg/kg diet)		3.750^ab^	6
Mn (84 mg/kg diet) ‐ P (0 mg/kg diet) ‐ ME (0 mg/kg diet)		3.250^abc^	4.75
Mn (84 mg/ mg/kg diet) ‐ P (200 mg/kg diet) ‐ ME (0 mg/kg diet)		3.250^abc^	4.75
Mn (84 mg/kg diet) ‐ P (0 mg/kg diet) ‐ ME (1,000 mg/kg diet)		3.250^abc^	5.25
Mn (84 mg/kg diet) ‐ P (200 mg/kg diet) ‐ ME (1,000 mg/kg diet)		4.250^a^	6.75
*SEM*		0.327	0.605
*p*‐value		0.026	0.2998

Note

ATAN1: antibody titre against Newcastle at first time; ATAN2: antibody titre against Newcastle at second time; ME: Multi‐enzyme; P: probiotic; *SEM*: standard error of the means.

The means within the same column with at least one common letter, do not have a significant difference (*p* > .05).

^a, b, c, d, e, f^ the means within the same row with different letter, are significantly different (*p* < 0.05).

Tables 5 and 6 show the results of two measurements of IgG, IgM and total immunoglobulin produced after SRBC (sheep red blood cells) injection. According to the results, the responses of immunoglobulin titres produced after the first stage of SRBC injection were not significantly different in any of the experimental groups (*p* > .05). In the second stage, IgM titres in the experimental groups containing manganese and IgG in the experimental groups containing probiotics had a significant increase compared to the control (*p* < .05). In the combined use, the difference in IgM and total immunoglobulin concentrations produced against SRBC in treatments containing manganese, probiotics and multi‐enzymes were significantly increased compared to other treatments (*p* < .05). The highest significant difference in IgG, IgM concentrations and total immunoglobulin produced against SRBCs were observed for the groups fed by 84mg manganese along with probiotics and multi‐enzymes compared to the control (*p* < .05).

**TABLE 5:** The effects of experimental diets on immune responses of broiler chickens after injection of the first stage of SRBC

Treatment		IgG (log_10_)	IgM (log_10_)	Total Ig (log_10_)
Mn (mg/kg diet)	0	0.625	1.063	1.688
	60	0.938	1.125	2.063
	72	1	1.125	2.188
	84	1.25	1.188	2.375
*SEM*		0.192	0.092	0.237
*p*‐value		0.1604	0.8197	0.2273
Probiotic (mg/kg diet)	0	0.813	1.125	1.938
	200	1.094	1.125	2.219
*SEM*		0.136	0.065	0.168
*p*‐value		0.1491	1	0.2418
Multi‐enzyme (mg/kg diet)	0	0.938	1.094	2.031
	1,000	0.969	1.156	2.125
*SEM*		0.136	0.065	0.168
*p*‐value		0.8713	0.5002	0.6946
Mn (0 mg/kg diet) ‐ P (0 mg/kg diet) ‐ ME (0 mg/kg diet)		0	1	1
Mn (0 mg/kg diet) ‐ P (200 mg/kg diet) ‐ ME (0 mg/kg diet)		0.75	1.25	1.75
Mn (0 mg/kg diet) ‐ P (0 mg/kg diet) ‐ ME (1,000 mg/kg diet)		0.5	1	1.5
Mn (0 mg/kg diet) ‐ P (200 mg/kg diet) ‐ ME (1,000 mg/kg diet)		1.25	1.25	2.5
Mn (60 mg/kg diet) ‐ P (0 mg/kg diet) ‐ ME (0 mg/kg diet)		0.5	1	1.75
Mn (60 mg/kg of diet) ‐ P (200 mg/kg of diet) ‐ ME (0 mg/kg diet)		1.25	1	2.25
Mn (60 mg/kg diet) ‐ P (0 mg/kg diet) ‐ ME (1,000 mg/kg diet)		0.5	1	1.5
Mn (60 mg/kg diet) ‐ P (200 mg/kg diet) ‐ ME (1,000 mg/kg diet)		1.25	1.25	2.5
Mn (72 mg/kg diet) ‐ P (0 mg/kg diet) ‐ ME (0 mg/kg diet)		0.5	1	2.5
Mn (72 mg/kg diet) ‐ P (200 mg/kg diet) ‐ ME (0 mg/kg diet)		1.25	1	1.5
Mn (72 mg/kg diet) ‐ P (0 mg/kg diet) ‐ ME (1,000 mg/kg diet)		1	1	3
Mn (72 mg/kg diet) ‐ P (200 mg/kg diet) ‐ ME (1,000 mg/kg diet)		1.5	1.25	2.75
Mn (84 mg/kg diet) ‐ P (0 mg/kg diet) ‐ ME (0 mg/kg diet)		0.75	1	1.75
Mn (84 mg/ mg/kg diet) ‐ P (200 mg/kg diet) ‐ ME (0 mg/kg diet)		1.25	1.25	2.25
Mn (84 mg/kg diet) ‐ P (0 mg/kg diet) ‐ ME (1,000 mg/kg diet)		1	1	2
Mn (84 mg/kg diet) ‐ P (200 mg/kg diet) ‐ ME (1,000 mg/kg diet)		2	1.75	2.75
*SEM*		0.384	0.184	0.475
*p*‐value		0.0941	0.283	0.171

Note

ME: Multi‐enzyme; P: probiotic; *SEM*: standard error of the means.

The means within the same column with at least one common letter, do not have significant difference (*p* > .05)

^a, b, c, d, e, f^ the means within the same row with different letter, are significantly different (*p* < 0.05).

**TABLE 6:** The effects of experimental diets on immune responses of broiler chickens after injection of the second stage of SRBC

Treatment		IgG (log_10_)	IgM (log_10_)	Total Ig (log_10_)
Mn (mg/kg diet)	0	2.5	2.125^c^	5.313
	60	3	2.750^b^	6.313
	72	2.688	2.813^b^	5.438
	84	3.063	3.313^a^	5.188
*SEM*		0.231	0.163	0.31
*p*‐value		0.2797	<0.0001	0.0558
Probiotic (mg/kg diet)	0	2.563^b^	2.781	5.344
	200	3.063^a^	2.719	5.781
*SEM*		0.163	0.116	0.219
*p*‐value		0.0355	0.7037	0.1652
Multi‐enzyme (mg/kg diet)	0	2.625	2.75	5.375
	1,000	3	2.75	5.75
*SEM*		0.163	0.116	0.219
*p*‐value		0.1111	1	0.2329
Mn (0 mg/kg diet) ‐ P (0 mg/kg diet) ‐ ME (0 mg/kg diet)		1.750^b^	1.750^d^	3.750^d^
Mn (0 mg/kg diet) ‐ P (200 mg/kg diet) ‐ ME (0 mg/kg diet)		3.000^b^	3.000^abc^	6.250^abc^
Mn (0 mg/kg diet) ‐ P (0 mg/kg diet) ‐ ME (1,000 mg/kg diet)		2.250^b^	2.500^bcd^	4.250 cd
Mn (0 mg/kg diet) ‐ P (200 mg/kg diet) ‐ ME (1,000 mg/kg diet)		3.000^b^	3.000^abc^	6.000^abc^
Mn (60 mg/kg diet) ‐ P (0 mg/kg diet) ‐ ME (0 mg/kg diet)		2.750^b^	2.500^bcd^	5.750^abcd^
Mn (60 mg/kg of diet) ‐ P (200 mg/kg of diet) ‐ ME (0 mg/kg diet)		2.750^b^	3.000^abc^	6.000^abc^
Mn (60 mg/kg diet) ‐ P (0 mg/kg diet) ‐ ME (1,000 mg/kg diet)		3.250^b^	2.750^abcd^	5.750^abcd^
Mn (60 mg/kg diet) ‐ P (200 mg/kg diet) ‐ ME (1,000 mg/kg diet)		3.250^b^	3.250^ab^	6.000^abc^
Mn (72 mg/kg diet) ‐ P (0 mg/kg diet) ‐ ME (0 mg/kg diet)		3.250^b^	2.250^bcd^	4.750^bcd^
Mn (72 mg/kg diet) ‐ P (200 mg/kg diet) ‐ ME (0 mg/kg diet)		3.000^b^	3.000^abc^	6.000^abc^
Mn (72 mg/kg diet) ‐ P (0 mg/kg diet) ‐ ME (1,000 mg/kg diet)		2.750^b^	2.500^bcd^	5.250^abcd^
Mn (72 mg/kg diet) ‐ P (200 mg/kg diet) ‐ ME (1,000 mg/kg diet)		1.750^b^	3.250^ab^	6.500^ab^
Mn (84 mg/kg diet) ‐ P (0 mg/kg diet) ‐ ME (0 mg/kg diet)		1.750^b^	2.000 cd	4.500 ^bcd^
Mn (84 mg/ mg/kg diet) ‐ P (200 mg/kg diet) ‐ ME (0 mg/kg diet)		2.750^b^	3.000^abc^	6.500^ab^
Mn (84 mg/kg diet) ‐ P (0 mg/kg diet) ‐ ME (1,000 mg/kg diet)		2.750^b^	2.500^bcd^	4.500^bcd^
Mn (84 mg/kg diet) ‐ P (200 mg/kg diet) ‐ ME (1,000 mg/kg diet)		5.000^a^	3.750^a^	7.250^a^
*SEM*		0.462	0.327	5.313
*p*‐value		0.0029	0.0105	6.313

Note

ME: Multi‐enzyme; P: probiotic; *SEM*: standard error of the means.

The means within the same column with at least one common letter, do not have significant difference (*p* > .05)

^a, b, c, d, e, f^ the means within the same row with different letter, are significantly different (*p* < 0.05).

## 6 DISCUSSION

The results of the present experiment showed that increasing the level of manganese significantly increased the weight of broilers in the entire period and improved the feed conversion ratio, which is consistent with the results of Sirri et al., (2016) who stated that manganese typically plays an important role in activating the enzyme systems effective in the metabolism of carbohydrates, fats, proteins, which can lead to a better use of dietary compounds and better weight gain, as well as improved feed conversion ratio in broilers. In line with the results of the present experiment, Pournazari et al., (2017) reported that broilers given diets containing enzymes or probiotics had a significant increase in live weight gain compared to the control and their conversion ratio was significantly reduced. One reason for the effectiveness of probiotics is their ability to inhibit the growth of harmful bacteria in the digestive system. In addition, bacteria in probiotics increase the activity of digestive enzymes. The release of metabolizable energy in the feed increases due to the effect of digestive enzymes on diet components. Thus, it increases the weight of broilers and improves their conversion ratio (Mountzouris et al., 2015).

Some researchers have reported that the use of multi‐enzymes in broiler diets has beneficial effects on weight gain and conversion ratio. According to them, the use of enzymes improves the digestibility of feed and increases the availability of the nutrients such as starch, protein, fat, etc., by reducing the viscosity of non‐starch polysaccharides and breaking bonds that are not digestible by the animal's internal enzymes. The use of enzymes in the diet also strengthens the enzymes present in the animal's body. This is especially important in young animals because the digestive system in young animals is not fully developed and the production of internal enzymes is usually not enough for digestion (Hasani et al., 2019).

Regarding the interacting effects of the combined uses, among the different experimental groups, the highest significant difference was observed for weight gain and the lowest significant difference was for conversion ratio in the diets containing 72 and 84 mg of manganese with probiotics and multi‐enzymes.

Little is known on the interactive effects of manganese, probiotics and multi‐enzymes in diets on broiler performance. In an experiment conducted on broilers by Jin et al., (2002), it was stated that the treatments containing mixed enzymes and probiotics had significant weight gain and improved conversion ratio compared to the control, which is consistent with the results of this experiment.

In the present experiment, the combined use of manganese with probiotics and multi‐enzymes in broiler diets increased performance (weight gain and improved conversion ratio) compared to the control and the other groups. Enzyme activity is affected by the pH of the digestive system. Most enzymes work at an acidic or neutral pH. Therefore, increasing the activity of the bacteria in probiotics and lowering the pH can help improve the digestion of nutrients in the intestine by means of the enzyme. On the other hand, when the body's ability to access nutrients increases, the manganese element can better play its role in improving performance because manganese can digest, absorb and convert carbohydrates and fats into energy with the help of nutrients (Aftab & Bedford, 2018; Brooks et al., 2012; Hajati et al., 2018).

The effect of different levels of manganese, as well as the use of probiotics and enzymes, was not significant on the average feed intake of chickens throughout the entire period. In addition, in the effects of combined uses, the feed intake of the experimental groups did not show a significant difference.

Although probiotics and enzymes used in the diet were able to reduce feed intake, this reduction was not significant, which is consistent with the results of Siadati et al., (2018). They stated that probiotics and enzymes, because of their specific properties, improve the availability of nutrients and their digestion, and may reduce the animal's need to consume more feed for obtaining nutrients (Attia et al., 2017; Siadati et al., 2018).

Our results show that the difference in the weight of the lymphoid organ of bursa of Fabricius had a significant increase in different levels of manganese compared to the control. Some studies have stated that a sufficient amount of manganese in the diet improves the immune system by increasing the antioxidant activity of superoxide dismutase (SOD) and removing more toxins from cellular metabolism (free radicals). Thus, its deficiency leads to changes and damage to the tissues of immune organs such as the bursa of Fabricius, which are the place where lymphocytes are produced, followed by dysfunction and reduced growth of the organ (Damaceno Faria et al., 2020; Ji et al., 2006).

How manganese, probiotics and enzymes interact is not clear but Majedi et al., (2015) reported that increasing relative weight gain of lymphoid organs is a sign of a strengthened immune system. Probiotics improve the immune system of birds and regulate their immune processes by destroying internal pathogens and the anatomical evolution of the lymphatic tissues of the thymus, bursa and spleen and other organs dependent on the immune system of broilers, as well as by enhancing mature defence cells.

According to Pourakbari et al., (2016) the lack of nutrients in the diet or lack of proper absorption of them in the intestinal tract can reduce the activity of the immune system and that improving digestion and absorption through the use of probiotics and enzymes can cause optimal safety because of access to materials required by the immune system. Wang et al., (2010) also reported that probiotics can strengthen the immune system by improving the utilization of the minerals such as manganese, which is one of the minerals needed to strengthen the immune system.

Consistent with this study on the increased response to antibodies produced against NDV in the diets containing probiotics and multi‐enzymes compared to controls, Scott et al., (1997) and Poorghasemi et al., (2017) observed by increasing digestibility the enzymes increase the bird's access to nutrients (proteins and carbohydrates) and probiotics by improving nutrient intake from the small intestinal wall and stimulating the growth of lymphoid follicles in primary and secondary lymphoid organs to produce and proliferate B and T cells, can improve humoral immune response.

According to previous studies, increasing the amount of manganese along with probiotics and multi‐enzymes, increased the levels of antibody produced against NDV. It is believed that manganese‐dependent superoxide dismutase in mitochondria activates the free radicals released inside the cells. The organic form of this mineral enhances the antibody response against the NDV vaccine in broilers. As it is a super oxidation cofactor, the need for manganese also increases during immune responses. Therefore, with sufficient manganese available in the body, the antibody titre will be higher and the immune system will be stronger, which may be the reason for the increased response to antibodies produced against NDV by increasing the level of manganese (Brooks et al., 2012).

In our study, all three levels of manganese were able to increase the concentration of IgM compared to the control. This is consistent with the hypothesis of increasing the antibody titre using manganese chelate and is similar to the works of researchers who stated that the organic form of minerals has a significant positive effect on the immune system. It has been reported that manganese chelate in broilers balanced T‐helper lymphocytes (Th1 and Th2), which play a role in activating macrophages and inducing antibody production; it was also shown that they increased the expression of M and G immunoglobulins (Burin Junior et al., 2018).

The probiotic groups showed a significant increase in IgG concentration compared to the control, which is consistent with the results of Khaksefifi and Ghoorchi (2006). These authors report that adding probiotics to poultry diets increases cellular growth and development of cavities or Peyer's patches of the ileum, which is a sign of stimulation of the mucosal immune system which reacts to antigenic stimuli through the secretion of immunoglobulins. They also stated that probiotics activate the immune system to counteract various antigens by increasing the xenophobia of lymphocytes and the production of mediated‐cytokines of T‐helper cells by lymphoid organ cells, which may increase IgG titres after SRBC injection in probiotic groups.

The level of antibody produced against NDV was increased in the combined uses by increasing the amount of manganese along with probiotics and multi‐enzymes. In the results of interacting effects of the combined uses, the groups fed with 84 mg manganese along with probiotics and multi‐enzymes showed the highest significant difference in IgG, IgM titres as well as total immunoglobulins produced against SRBC. Little research has been done on the interacting effects of manganese, probiotics and multi‐enzymes on the titres of immunoglobulins produced after SRBC injection. However, probiotics have been shown to stimulate different cells of the immune system to produce cytokines that are involved in inducing and regulating immune responses (Christensen et al., 2002; Hussein et al., 2020). Also, the animal body considers the entry of probiotic bacteria into the digestive system as an external organism and stimulates the immune system against these microorganisms and increases the animal's immunity (Panda et al., 2000). Enzymes reduce the viscosity of digestive substances by breaking large molecules of non‐starch soluble polysaccharides into small polymers and improving the immune status of birds by improving the absorption of micronutrients such as manganese (Bedford, 2009).

## 7 CONCLUSIONS

In general, the results of the present experiment show that the simultaneous use of manganese, probiotics and multi‐enzymes has additional beneficial effects on the performance and stimulation of the immune system of broilers and it can be produced by supplementing poultry feed with manganese, probiotics and multi‐enzymes, birds with high yield and strong immune system against diseases.

## CONFLICT OF INTEREST

The authors declare that they have no conflict of interest.

## AUTHORS' CONTRIBUTION

All authors contributed to and approved the final manuscript. Mohammad Javad Babaei: Conceptualization; Data curation; Investigation; Methodology; Project administration; Supervision; Writing‐ original draft; Writing‐review & editing. Jafar Fakhraei: Conceptualization; Data curation; Formal analysis; Investigation; Methodology; Project administration; Supervision; Writing‐ original draft; Writing‐review & editing. Hossein Mansoori Yarahmadi: Data curation; Investigation; Project administration; Writing‐original draft; Writing‐review & editing. Masoud Gomarian: Conceptualization; Data curation; Formal analysis; Investigation; Methodology; Project administration; Resources; Supervision; Validation; Visualization; Writing‐original draft; Writing‐review and editing.

## STATEMENT OF ETHICAL CONSIDERATION

The experimental protocol was ratified by the Animal Ethic Committee of the Arak Branch, Islamic Azad University, Arak, Iran and the experiment was performed with respect to the International Guidelines for research involving animals (Directive 2010/63/EU).

### PEER REVIEW

The peer review history for this article is available at https://publons.com/publon/10.1002/vms3.479.

## Data Availability

I certify that all data in the article are true and reliable. All public data generated or analysed during this study are included in this article. Data sharing is not applicable to this article as no new data were created or analysed in this study.
